# Pass/fail decisions and standards: the impact of differential examiner stringency on OSCE outcomes

**DOI:** 10.1007/s10459-022-10096-9

**Published:** 2022-03-01

**Authors:** Matt Homer

**Affiliations:** School of Medicine, Leeds Institute of Medical Education, University of Leeds, LS29JT Leeds, UK

**Keywords:** Examiner stringency, OSCE, Standard setting, Borderline regression

## Abstract

Variation in examiner stringency is a recognised problem in many standardised summative assessments of performance such as the OSCE. The stated strength of the OSCE is that such error might largely balance out over the exam as a whole. This study uses linear mixed models to estimate the impact of different factors (examiner, station, candidate and exam) on station-level total domain score and, separately, on a single global grade. The exam data is from 442 separate administrations of an 18 station OSCE for international medical graduates who want to work in the National Health Service in the UK. We find that variation due to examiner is approximately twice as large for domain scores as it is for grades (16% vs. 8%), with smaller residual variance in the former (67% vs. 76%). Combined estimates of exam-level (relative) reliability across all data are 0.75 and 0.69 for domains scores and grades respectively. The correlation between two separate estimates of stringency for individual examiners (one for grades and one for domain scores) is relatively high (r=0.76) implying that examiners are generally quite consistent in their stringency between these two assessments of performance. Cluster analysis indicates that examiners fall into two broad groups characterised as hawks or doves on both measures. At the exam level, correcting for examiner stringency produces systematically lower cut-scores under borderline regression standard setting than using the raw marks. In turn, such a correction would produce higher pass rates—although meaningful direct comparisons are challenging to make. As in other studies, this work shows that OSCEs and other standardised performance assessments are subject to substantial variation in examiner stringency, and require sufficient domain sampling to ensure quality of pass/fail decision-making is at least adequate. More, perhaps qualitative, work is needed to understand better how examiners might score similarly (or differently) between the awarding of station-level domain scores and global grades. The issue of the potential systematic bias of borderline regression evidenced for the first time here, with sources of error producing cut-scores higher than they should be, also needs more investigation.

## Introduction

It is well known that the impact of variation in examiner stringency is a threat to the validity of OSCE-type assessment outcomes (Bartman et al., 2013; Harasym et al., 2008; McManus et al., 2006; Yeates et al., 2018; Yeates & Sebok-Syer, 2017). In larger OSCEs, the assessment design means that candidates are usually grouped in parallel circuits and ‘see’ a specific set of examiners (Khan et al., 2013; Pell et al., 2010), which means that it is very difficult to disentangle examiner effects from differences in candidate ability (Yeates et al., 2021; Yeates & Sebok-Syer, 2017). In a single administration of a small OSCE there might be a unique set of examiners for each cohort of candidates, but across different exam administrations the same issues of unwanted variation in scores due to examiner stringency arises. This problem, often referred to colloquially as a ‘hawks and doves’ effect (McManus et al., 2006; Yeates & Sebok-Syer, 2017), is particularly a concern at the station level. The stated strength of the OSCE, however, is that such error might largely balance out over the exam as a whole through wide sampling of examiners and stations, and via attempts to standardise the overall assessment process through, for example, structured scoring instruments, and appropriate examiner training (Harden et al., 2015, Chapters 9 and 11). However, some research brings into question the assumption that scoring error largely cancels out at the exam level (Homer, 2020; Yeates et al., 2018, 2021).

OSCEs are typically scored in two different ways—using a checklist or domain scoring to capture specific aspects of the encounter, and then a holistic grade intended to capture the quality of the overall performance (Homer et al., 2020; Khan et al., 2013). Most of the literature on examiner stringency has focused analysis of differences in the former, and there has been little empirical work looking at how individual examiner stringency might compare between the awarding of global grades and of checklist/domain scores at the station level in an OSCE (or similar). In a US workplace clerkship setting Santen and colleagues (2021) show that variance due to students at the item/domain level was small (4-8%) and that due to rater (nested in student) was much higher (of the order of 50%).One study that did include checklists and global grades (by Jogerst and colleagues (2021)) found that the rating of technical skills between pair of raters was inconsistent, but did not directly compare differences in stringency of individual raters on their checklist and global scoring.

This study uses candidate-level data from a large number of administrations of an OSCE where examiners and stations are repeatedly employed over a three year period. The nature of the data allows the disentanglement of examiner and station effects, and the comparison of estimates of examiner stringency in domain scoring and global grades.

The assessment context is the PLAB2 OSCE, an 18 station summative assessment for international medical graduates who want to begin working in the National Health Service in England (General Medical Council 2020a, 2020b). The level of the exam is set at that appropriate for a post-graduate trainee entering the second year of training (called FY2 in the UK). There is a single examiner in each station, and in most stations there is a simulated patient (SP) played by a paid actor. Quality is assured through a range of examiner training, pre-exam calibration between examiners and SPs, a range of post hoc analysis, and general exam oversight by a highly experienced assessment panel of 30-40 senior clinicians. The assessment is intended to ensure that candidates can apply their medical knowledge in order to provide good care to patients at the FY2 level. For more details of this particular assessment context see for example Homer et al. (2019). Analysis of station-level data in this setting (i.e. all candidate level data aggregated to station level) indicates that examiner variation in station-level standards can be accounted for by appropriate use of the standard error of measurement (Hays et al., 2008; Homer, 2020). This paper provides a more fine-grained analysis that includes candidate effects using fully anonymised candidate-level data from the same setting.

This study also investigates overall pass/fail decision making under the borderline regression method of standard setting (Kramer et al., 2003; McKinley & Norcini, 2014; Pell et al., 2010) having adjusted for differences in station difficulty and examiner stringency across administrations for both domain scores and global grades.

In terms of specific research questions, we set out to answer the following:

1. What are candidate, examiner, station and exam effects on total domain scores and global grades?
2. How do the two estimates of examiner stringency (one each for domain scores and global grades respectively) compare—to what extent are examiners consistent in their stringency across these?
3. What is the impact on station cut-scores and overall pass/fail decisions of the adjustment of candidate scores and grades to ‘fair’ scores?

We also review the limitations of this type of study, and consider the implications of the findings for wider OSCE practices.

In the next section of the paper we provide an overview of the data and statistical methods employed, and then the present the key findings of the research. The paper concludes by considering the implications of this study, and areas for further research.

## Samples and methods

### Examinations and facets

The original data consisted of 75% candidate first attempts at PLAB2, and another 20% as second attempts (with, typically, and improved performance on second attempt). A data preparation process was carried out to allow for the inclusion these second attempts. This maximises the use of the available data with 95% of available candidate level data then included. Full details of this process are given in Appendix 1). This process produced a final dataset containing 313,593 candidate/station interactions from 442 PLAB2 examinations over the period November 2016 to March 2020. Table 1 summarises the frequency of occurrence of all main facets in the data used in this paper.

**Table 1:** Descriptive statistics for the key facets of the PLAB2 exam

Facet	Number of unique levels (i.e. values) in data	Typical occurrence in data		
		**Median (quartiles)**	**Mean**	**Description**
Candidates	17,604	18 (18,18)	17.8	Typically candidates are assessed at 18 stations in PLAB2. Occasionally, a station might be removed from the examination due to poor psychometric performance.
Examiners	862	6 (3,13)	11.1	Typically examiners are present in six PLAB2 exams in the dataset
Stations	390	17 (8, 29)	20.2	Typically stations are administered in 17 exams in this dataset
Exams	442	1 (1, 1)	1.0	The data is from 442 separate PLAB2 exams
Observations	313,593	Not applicable	Not applicable	There are 313,593 rows of data—one for each candidate/station interaction.

### The two assessment outcomes

The outcomes awarded by the single examiner in each PLAB2 station are:

**An overall domain score** on scale from 0 to 12. This is the sum of three separate domain scores (each scored 0 to 4) in (i) *Data gathering*, *technical and assessment skills*, (ii) *Clinical management skills*, and (iii) *Interpersonal skills*.

**A single global grade** on a scale from 0 to 3 (0 = fail, 1 = borderline, 2 = satisfactory, 3 = good), providing an overall holistic judgment of candidate performance in the station.

**Fig. 1:**
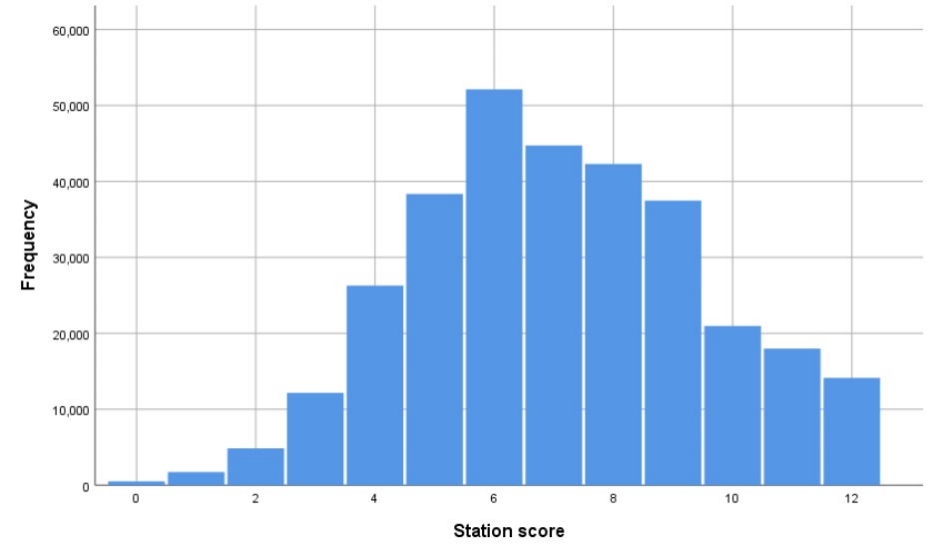
Histogram of station scores (n = 313,593)

**Fig. 2:**
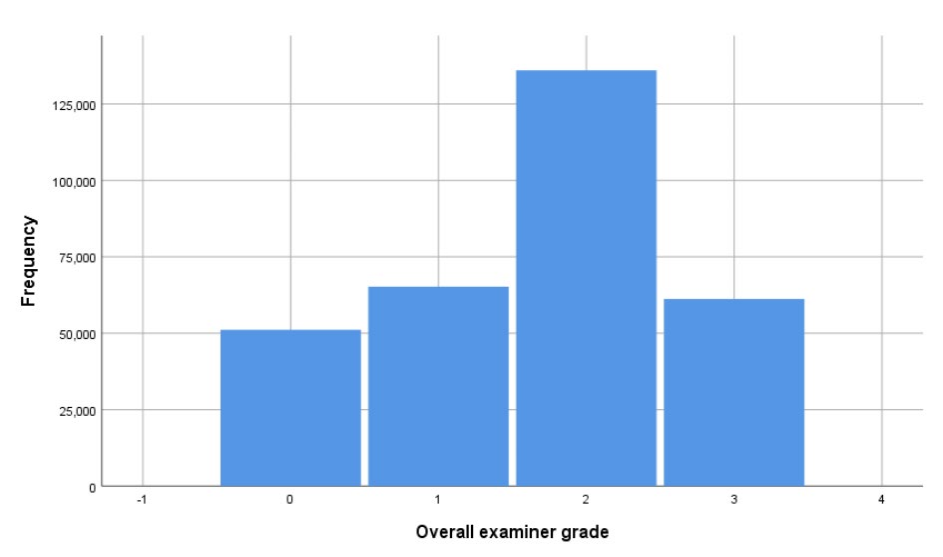
Histogram of global grades (n = 313,593)

Figures 1 and 2 show the distributions of these two assessment outcomes in the full dataset—these are the outcomes that are modelled by the random facets shown in Table 1 (more on this in the next section).

The correlation between these two station-level estimates across all data is 0.847 (p < 0.001, n = 313, 593). Note, this correlation will be weakened by measurement error, which has not been adjusted for here or in later analyses.

### Main modelling approach—for total domain score and global grade (RQ1)

To answer RQ1, we use linear mixed models in the R package lme4 (Bates et al., 2015) to separate out variance in station-level domain scores due to candidate, station, examiner and exam facets (Table 1), treating each of these as random effects:

**Table 2:** Outline of main modelling approach for domain scores

Domain score modelled by random effects of *Candidate*, *Station*, *Examiner* and *Exam* In pseudo-code the equation is:
DOMAIN_SCORE ~ 1 + (1 | CANDIDATE) + (1 | EXAMINER) + (1 | STATION) + (1 | EXAM) (the notation (1| FACET) indicates it is being treated as a random effect)

Interaction effects between these facets are not estimable in the data available. For example, each candidate is present only once within the data, and whilst examiners are present for a number of station administrations, it is rare for the same examiner to assess in the same station more than once (Table 1). This is a limitation we return to in the Discussion.

We estimate a second model, with identical facets as random effects (Table 1), but with global grades instead of domain scores as the dependent variable. See Appendix 2 for details of the model-checking for both models.

We use generalisability theory (Bloch & Norman, 2012; Brennan, 2001) to estimate overall measures of reliability (both relative and absolute), and the standard error of measurement—using the variance components produced by the linear mixed modelling (Jiang, 2018).

### Measures of stringency and cluster analysis (RQ2)

In addition to variance components for each facet, the modelling produces separate estimates for *Candidate*, *Station*, *Examiner* and *Exam* stringency for each level of the facet—so for each individual candidate, station and so on. The following gives a description of how these are best interpreted:

**Table 3:** Interpreting stringency estimates

Facet	Interpretation of individual model estimate
*Candidate*	The expected outcome for the candidate in a typical station, with a typical examiner and typical exam. This is therefore a single measure of candidate ‘ability’ having taken account of all other facets—so can be thought of as an estimate of the ‘fair’ score for the candidate.
*Station*	The expected outcome at this station for a typical candidate examined by a typical examiner in a typical exam. This is an estimate of station difficulty having taken account of all other facets, with easier stations having higher values.
*Examiner*	The expected outcome awarded by the examiner who assesses a typical candidate at a typical station in a typical exam. This is an estimate of examiner stringency having taken account of all other facets, with more hawkish examiners having lower values.
*Exam*	The expected outcome for the exam, assuming a typical set of candidates, stations and examiners. This is a measure of exam difficulty having taken account of all other facets, with easier exams having higher values.

We then use correlation to estimate the strength of the relationships between various model outcomes—in particular, to compare observed candidate performance with modelled-based ‘fair’ scores, and to compare estimates of examiner stringency between domain scores and global grades.

#### Cluster analysis

We use the estimates of examiner stringency in domain scores and global grades to help answer RQ2 by carrying out a relatively simple two-step cluster analysis (Bacher et al., 2004) to investigate patterns of examiner behaviour on these two measures.

### Examination level pass/fail decisions (RQ3)

A key element of this research (RQ3) is to better understand at the exam level, what the impact of examiner stringency might be having on candidate pass/fail outcomes. Hence, we compare overall candidate outcomes between those observed and those from the modelling, in a context where borderline regression standard setting is used (Kramer et al., 2003; McKinley & Norcini, 2014; Pell et al., 2010). In borderline regression, station-level checklist or domain scores are regressed on global grades, and the cut-score is usually set at the predicted value at the borderline grade. The cut-score at the exam level is the aggregate of the station-level cut-scores (McKinley & Norcini, 2014).

## Findings

### Sources of variation and reliability of scores and grades (RQ1)

Table 4 shows the partitioning of the variance in station level outcomes using linear mixed model for domain scores, and then separately for global grades, both based on random effects of *Candidate*, *Station*, *Examiner* and *Exam* (Table 2).

**Table 4:** Variance in station-level scores and grades from separate linear mixed models (n = 313,593)

Facet	Model for domains scores		Model for global grades	
	**Variance**	**Percentage**	**Variance**	**Percentage**
Candidate	0.685	11.4%	0.091	9.5%
Station	0.347	5.7%	0.060	6.3%
Examiner	0.958	15.9%	0.075	7.9%
Exam	0.030	0.5%	0.004	0.4%
Residual	4.013	66.5%	0.726	75.9%
**Total**	**6.032**	**100.0%**	**0.956**	**100.0%**

Focussing first on domain scores, Table 4 shows that there is more variance due to *Examiner* at the station level than other facets, but that two-thirds of variance is not explained by any of these facets (residual 66.5%).

The story for global grades is a little different, as the *Candidate* facet account for a higher percentage of variance than does *Examiner* (9.5% vs. 7.9%). However, the amount of residual variance is higher when comparing with domain scores (75.9% vs. 66.5%).

For both outcomes, the *Station* facet accounts for a relatively small proportion of variance (5.7% and 6.3% for scores and grades respectively), and the *Exam* facet for an even smaller amount (0.5% and 0.4%).

Within a generalisability framework, we can use the variance components from Table 4 to calculate overall reliability coefficients and standard errors of measurement (SEM) for a typical single 18 station PLAB2 exam - Table 5.

**Table 5:** Overall reliability/SEM estimates for an 18 station PLAB2 OSCE

	Statistic	Domain scores (12 point scale)	Global grades (3 point scale)
**Relative**	**Generalizability coefficient (G)**	0.754	0.692
	**SEM**	0.472 (3.93%)	0.201 (6.69%)
**Absolute** (treating all non-candidate variance as error)	**Dependability coefficient (Phi)**	0.678	0.635
	**SEM**	0.571 (4.76%)	0.228 (7.60%)

The value of these estimates are acceptable according to the usual guidelines (Lance et al., 2006; Park, 2019), but as a result of the greater residual variance for grades (Table 4), the reliability estimate for global grades are lower, and the SEMs correspondingly greater, than they are for domain scores.

#### Correlations between observed and modelled station-level scores and grades

Table 6 shows the (Pearson) correlation coefficients for domain scores and grades—for both observed values (i.e. those actually produced by examiners) and modelled values derived in the linear mixed modelling.

**Table 6:** Correlation between observed and modelled values across all candidate/station interactions

Pearson correlation coefficient (n=313,593, all p < 0.001)	Observed domain score	Observed global grade	Modelled domain score
**Observed global grade**	0.85		
**Modelled domain score**	0.60	0.45	
**Modelled global grade**	0.53	0.52	0.86

The overall correlation between observed scores and grades is strong overall (r = 0.85), and is very similar to that between the modelled values of these (0.86). Importantly, the correlation between observed and modelled values are not as strong—r = 0.60 for domain scores, and 0.52 for global grades. This indicates that the modelling has actually had an important impact on adjusting scores/grades when ‘controlling’ for unwanted sources of variance (*Station*, *Examiner* and *Exam*).

### Individual modelled estimates of stringency of each facet (RQ2)

The modelling gives summary statistics for *Candidate*, *Station*, *Examiner* and *Exam* stringency for all levels of the facet (see Table 3 for more details on how to interpret each of these).

**Table 7:** Summary statistics for estimates of stringency for each facet (station-level)

Facet	Domains scores (12 point scale)		Global grades (3 point scale)	
	**Mean (SD)**	**Quartiles: Q1, median, Q3**	**Mean (SD)**	**Quartiles: Q1, median, Q3**
**Candidate** (n = 17,604)	7.24 (0.71)	6.77, 7.25, 7.70	1.66 (0.25)	1.50, 1.68, 1.83
**Station** (n=390)	7.24 (0.58)	6.91, 7.29, 7.67	1.66 (0.24)	1.52, 1.69, 1.84
**Examiner** (n = 862)	7.24 (0.96)	6.54, 7.22, 7.87	1.66 (0.26)	1.50, 1.67, 1.84
**Exam** (n = 442)	7.24 (0.13)	7.15, 7.23, 7.33	1.66 (0.05)	1.63, 1.66, 1.69

Table 7 shows that the mean values for each facet are the same—this is a natural consequence of the modelling. More importantly, by comparing standard deviations, the modelling suggests that there is greater variation in examiner stringency than there is for candidate ability, and this is particularly the case for domain scores (SD = 0.96, 0.71 respectively). In addition, variation in station difficulty is of a smaller magnitude, and variation across exams is very small. All these results are entirely consistent with the variance component analysis in Table 4.

#### Cluster analysis of examiner stringency

The correlation between the two estimates of examiner stringency (for domain scores and global grades) is quite strong at r = 0.76 (n = 862, p < 0.001) indicating that examiners are broadly consistent in their level of stringency across the two methods of scoring performance in a station. Taking the analysis further, a simple cluster analysis results in a two-cluster solution with ‘fair’ fit (silhouette score = 0.6, (Norusis, 2011, Chapter 17)). This is the maximum number of clusters when only two variables (examine stringency in domain scores and grades) are present in the analysis.

**Fig. 3:**
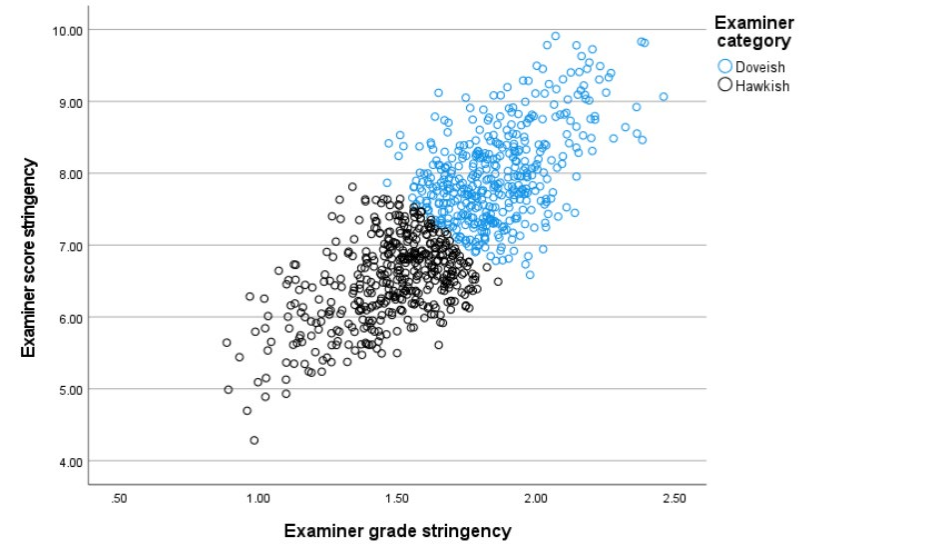
Scatter graph of the two estimates of examiner stringency with cluster allocation (n = 862)

Figure 3 shows a scatter graph of the two sets of estimates, with clusters labelled as hawkish (for those estimated as scoring relatively lowly) and doveish (for those estimated scoring more highly).

### Standard setting comparisons at station and exam level (RQ3)

We can compare borderline regression method (BRM) cut-scores derived from observed scores/grades in a station with those derived from the modelled outcomes. This provides us with insight into how the combined effect of examiner stringency (i.e. in both scores and grades) impacts on BRM standards, and then on candidate pass/fail outcomes. We note that in practice, cut-scores in PLAB2 are higher, and subsequent pass rates lower, than those presented here. For reasons of simplicity and data comparability, the comparison in this study is kept straightforward, omitting some elements of the actual standard setting approach—Appendix 3 gives more justification for this decision.

With 442 exams and 17.8 stations per exam (Table 1), there are a total of 7,877 separate station administrations in the data. The key overall finding when comparing these cut-scores, at both the station and the exam level, is that those derived from modelled outcomes are systematically lower than those based on the actual observed scores. To our knowledge, this is a completely new finding that has not been evidenced before in the literature.

At the station level, a paired t-test gives a mean difference between cut-scores from observed and modelled data of around 3% on the 12 point scale (mean observed = 5.61, mean modelled = 5.24; t = 57.94, df = 7876, p < 0.001, Cohen’s d = 0.65).

The equivalent analysis at the exam level ( ) gives a similar mean difference in exam level percentage scores of 3.1% (mean observed = 46.76, mean modelled = 43.66; t = 52.68, df = 441, p < 0.001, Cohen’s d = 2.51). The larger Cohen’s d at the exam level is a result of a much smaller (relative) standard deviation (of the difference) at this level. This lower SD is an artefact of the summing of a set of station-level cut-scores, a set of 18 somewhat independent random variables.

Additional analysis indicates that the average difference in cut-scores is a direct result of the systematic differences between BRM intercepts and slopes derived from modelled and observed data—as a result of error being removed during the modelling process. In short, regression slopes are typically higher in modelled data, and intercepts lower. Appendix 4 gives more a more detailed explanation of why these differences occur.

**Fig. 4:**
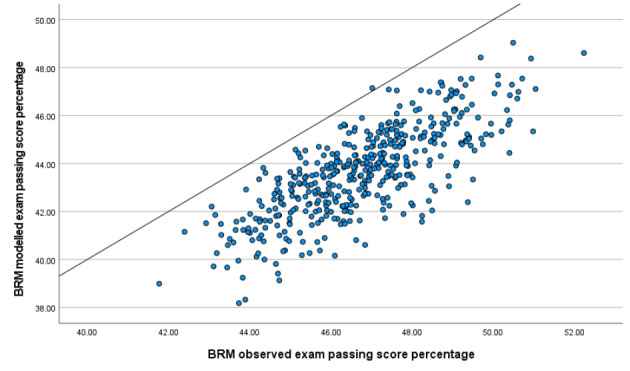
Scatter graph of exam-level observed and modelled exam passing score (percentage, n = 442)

Figure 4 shows a scatter graph of the two exam-level cut-scores (observed and modelled), with the line of equality shown. Almost all exam-level cut-scores are higher in the observed data than in that modelled.

That cut-scores are systematically lower once error has been removed was not anticipated, and one we will revisit in the *Discussion*.

Whilst this analysis has shown that cut-scores are systematically different between observed and modelled values, it should be emphasised that candidate domain scores (or grades) themselves do not differ on average. This is because residuals (= observed—modelled) are estimated with mean zero.

### Indicative differences in exam-level decisions (RQ3)

For completeness, the final analysis is a comparison of pass rates between observed and modelled data. However, as earlier caveats have indicated, this is quite problematic in some regards, and does not directly correspond to actual PLAB2 decision-making (again, see Appendix 3 for more on this).

Table 8 compares indicative exam-level pass/fail decisions between observed and modelled data.

**Table 8:** A hypothetical comparison between observed and modelled pass/fail decision

Overall candidate decisions in PLAB2		Pass Modelled		Total
		**No**	**Yes**	
**Pass Observed**	**No**	512	1750	2,262
	**Yes**	0	15,342	15,342
**Total**		512	17,092	17,604

As a result of the difference in exam-level cut-scores, the pass rate is much higher when using modelled data (97.1% vs. 87.2% for observed data).

For all the reasons already stated, this final analysis should be treated as indicative rather than representing a complete picture of how PLAB2 decision-making might change if scores were to be adjusted from observed to modelled in the way presented. We return to some of these issues in the *Discussion*.

## Discussion

### Examiner stringency—domains scores versus a global grade

This study uses a large-scale OSCE dataset to estimate separate influences of *Candidate*, *Examiner*, *Station* and *Exam* on station and overall performance, and to add to the very limited literature comparing estimates of examiner stringency in two different ways—via domain scores and global grades.

Overall, the results suggest that examiner stringency impacts more on station-level domain scores than on the single global grade (Table 4) but, accounting for other sources of error, the former, when summed, are more reliable than the latter (Table 5). The evidence in this area is mixed terms of which types of scoring instruments are most valid, but mostly compares checklists with global ratings of performance (Ilgen et al., 2015; Wood & Pugh, 2019). Santen and colleagues (2021) show that aggregating item-level scores can increase reliability at the station-level, and this is consistent with our findings. More work is needed to develop better understanding of the advantages and disadvantage of domain scoring in comparison to a single global rating of performance.

It is worth emphasising that there is a methodological innovation in this approach to calculating overall reliability across many exams (Jiang, 2018). For a single exam, it would only be possible to include *Candidate* and *Examiner* facets —*Station* is confounded with *Examiner* in single set of exam data, and obviously *Exam* only has one value. When outcomes from many exams are available, as with the PLAB2 data in this study, it becomes possible to separate out the impact of various facets (Table 2) on assessment outcomes. However, there remain limitations to this approach—for example *Candidate/Station* interactions, that is instances of case specificity (Norman et al., 2006), are not estimatable in this data as candidates only sit a particular station once (see Appendix 1) and examiner/station interactions are rarely repeated.

This study confirms that examiner stringency is a very important influence on station-level scoring/grading (Tables 4 and 7), and that adjusting for this does impact on station-level scores (Table 6). These findings are consistent with a wide range of literature (Homer, 2020; McManus et al., 2006; Santen et al., 2021; Yeates et al., 2018, 2021), but our work suggests that acceptable levels of overall assessment reliability can be achieved provided the number of stations is large enough (Table 5)—again consistent with other empirical and/or psychometric work (Bloch & Norman, 2012; Park, 2019). There is a lot of residual variance at the station level, and these results do suggest, however, that a focus on exam-level, rather than station-level, performance of a candidates is likely to be more meaningful in terms of good decision-making. Again, more work is needed to assess how appropriate the use of conjunctive standards such as minimum station hurdles is in these settings (Ben-David, 2000; Homer & Russell, 2020).

A key finding of this study is that examiners tend to be fairly consistent in their stringency when awarding different types of assessment outcomes (Fig. 1). It seems there is little specific literature in this area, and it might be valuable to further investigate qualitatively the different processes of how and why examiners might award grades and scores differently (Malau-Aduli et al., 2021; Tavares & Eva, 2013). Under examinee-centred methods of standard setting (e.g. BRM where scores/grades awarded are used to calculate cut-scores *post hoc)*, separate estimates of stringency across both scoring and grading is required to be able to re-calculate changes in cut-scores when assessing the impact on subsequent pass/fail decisions.

### A systematic bias in borderline regression?

The most unexpected finding in this study is that BRM cut-scores from observed scores/grades are systematically higher than the modelling suggests they should be—of the order of 3% at both station and exam level. This is mainly a direct consequence of variation in examiner stringency, which introduces error and directly impacts on the BRM standard via systematic effects weakening the slope and increasing the intercept of the regression line (Appendix 4). The methodological approach taken in this work suggests these results are unlikely to be unique to this PLAB2 context—many OSCEs must have a similar issue of systematic such differences in standards. It must be the case that in the presence of measurement error (e.g. because of examiner variation in stringency), borderline regression slopes will be lower than otherwise. However, it is very important to state that it is not clear whether in different contexts the impact of the error could go in the opposite direction i.e. that observed cut-scores could be lower than they would be were examiner (and other) error removed. In contexts similar to PLAB2, where the cut-score is set at a point relatively low in the ability range of candidates, it seems likely the impact on standards will be similar to that demonstrated here in PLAB2. However, more research is needed to develop understanding of these technical, but potentially impactful, issues across different contexts—perhaps using simulation methods (Morris et al., 2019). In PLAB2, our work suggests that healthcare consumers in the UK have greater protection as a result of the error that typically increases the required standard. For this reason, it is not currently envisaged that changes will be made to standard setting practices in PLAB2 as a consequence of the findings of this work.

### Study limitations and final conclusions

An obvious limitation of this study is that it is situated in a single examination context—ideally, the key findings need replication or a degree of confirmation elsewhere. Another potential problem is that there is a degree of artificiality about modelled scores/grades which is likely to inhibit their use in actual decision-making. One aspect of this is the treatment of the single ordinal global grade in each station as scale in the modelling, although this is exactly how such grades are treated under borderline regression (McKinley & Norcini, 2014). Another issue relates to the perfect reliability of modelled scores (i.e. reliability is 1, and standard error of measurement 0). This is a result of all hypothesised error having been removed, but it is very likely that there are other sources of error that have not been accounted for—for example, to do with interactions between different facets which cannot be estimated in this data. In the context of the assessment of international medical graduates such facets could be quite important (e.g. candidate/assessor or candidate/patient interactions). We argue that the statistical methods used here are valuable in quantifying error and its impact on the exam overall, but can never be truly confident that all sources of error have been captured and accounted for properly. This in turn implies that adjusting candidate-level scores and using these for actual decision-making is hard to justify, as has been argued elsewhere (Homer, 2020).

This study set out to investigate differential examiner stringency across the awarding of station-level domain scores and a single global grade, and found that these tend to be quite similar. It demonstrated that, under borderline regression methods, examiner error in PLAB2 leads to systematically higher cut-scores. More work is needed to better understand examiner behaviour when scoring performance, and to work through how these findings can inform the improvement of assessment policy in terms of, for example, the use of conjunctive standards (e.g. the addition of SEMs to cut-scores, and minimum station hurdles).

## Appendix 1

### Data perpetration - the treatment of non-first attempts

The original PLAB2 assessment data available for this study consisted of 338,197 candidate/station interactions from 454 examinations over the period November 2016 to March 2020. Approximately 75% of these interactions were from candidate first attempts, 20% were second attempts and the remainder from third or more attempts.

An initial analysis confirmed that candidates improved their overall scores significantly on average on a second attempt. A decision was therefore made to include second candidate attempts in this analysis, but to treat them as a different candidate in comparison to their first attempt—as in a key part of the modelling, the ability of the candidate is estimated and second attempts do not typically equate with first attempts. This decision maximises use of the original data (95% included), and, importantly, allows for meaningful examination-level analysis (i.e. of overall pass/fail decisions) without seriously undermining the nature of the data at this level (i.e. avoiding a relatively high proportion of missing candidates at the exam level). Candidate attempts other than first or second were removed.

In addition, 12 examinations were removed where candidate numbers were not equal across all stations in the exam.

## Appendix 2

### Model checking

As with all regression modelling, each model produces a predicted value for each observed outcome, and therefore a corresponding residual (observed value—modelled value). The residuals for the two linear mixed models are summarised in Table 9.

**Table 9:** Summary statistics for the linear mixed model residuals (station-level)

	Mean	Median	SD	Skew	Lower quartile	Upper quartile
**Domain scores** (12 point scale)	0	− 0.01	1.96	− 0.04	− 1.30	1.32
**Global grades** (3 point scale)	0	0.10	0.83	− 0.30	− 0.58	0.60

The residuals are broadly normal, which is a key modelling requirement, but with greater deviation from normality for the global grades than for the domain scores.

A large residual for a particular observation or set of observations does not necessarily imply a poor model. The models assumes a single candidate ‘ability’, and so cannot account for differential candidate performance across stations, for example, due to case specificity (Norman et al., 2006). Rather, the focus of this work is on adjusting overall candidate estimates of performance at the examination level in order to better understand the impact of examiner stringency at that level.

## Appendix 3

### Actual PLAB2 outcomes and pass rates

There are three main reasons why the cut-scores employed in PLAB2 are higher than those reported in this paper. Corresponding pass rates are lower than those reported.

1. A standard error of measurement (Hays et al., 2008) is added to the exam-level cut-score, but for simplicity this has not been included in the analysis.
2. Over the period of this study, a decision was made to increase the BRM standard (by using borderline = 1.2 instead of 1). In order to keep all data directly comparable, this change has not been included in this study.
3. A conjunctive standard (minimum stations passed) is also required to pass PLAB2, and again for reasons of simplicity this aspect has not been included in this analysis. This, in particular, is an area that requires further specific research.

Note that third and later attempts at PLAB2 have been removed from this study (Appendix 1)—these candidates scores would also contribute to standard setting and pass rates.

## Appendix 4

### Explaining the systematic difference in cut-scores between observed and modelled data

At the station level, the cut-score comprises the intercept plus the slope of the regression line derived during the BRM process (x = 1 corresponds to borderline). Additional analysis reveals that the slope is systematically lower, and the intercept higher, in the observed data compared to the modelled data. It is well known that measures with error in them correlate less strongly with each other compared with when the error is removed (Trafimow, 2016). For BRM, this means we would expect exactly the systematic difference in the slopes as just described. Because the modelling is overall neutral in terms of predicted values, this in turn means the intercepts must have the opposite pattern—intercepts are higher in observed compared to modelled data.

Using the mean values of these parameters across all station administrations (n = 7,877) Table 10 shows the predicted BRM regression line values. We can think of this as an average BRM line across all data that illustrates the average effect on cut-scores of error in scores/grades.

**Table 10:** Mean station BRM intercepts, slopes and predicted values (n = 7,877)

Grade (x value)	Description -	Predicted y-value (12 point scale)		Difference = Observed—modelled	
		**Observed**	**Modelled**	**On 12 point scale**	**Percentage**
0	Fail	3.55	2.51	1.04	8.6
1	Borderline	5.61	5.24	0.37	3.1
2	Satisfactory	7.68	7.97	− 0.29	− 2.4
3	Good	9.74	10.70	− 0.96	− 8.0

The second data row of Table 10 confirms the 3% difference shown in the paired t-test results in the main text. The average impact of error on cut-scores exemplified in Table 10 could in theory go in either direction, and may well be different in different contexts.
